# Supplementary material for: Quantifying the real-world impact of antibiotic use and genetic determinants of resistance on gonococcal dynamics
Source: Nat Microbiol. 2026 Jan 30;11(2):375–90. doi: 10.1038/s41564-025-02235-w (PMC12872460; doi:10.1038/s41564-025-02235-w)
Supplement: Supplementary file 1 — Supplementary Figs. 1–22 and Tables 1–9. [file 41564_2025_2235_MOESM1_ESM.pdf]

nature microbiology

Supplementary information https://doi.org/10.1038/s41564-025-02235-w

Quantifying the real-world impact of antibiotic use and genetic determinants of resistance on gonococcal dynamics

In the format provided by the authors and unedited

Supplementary materials

Figs. S1 to S22
Tables S1 to S9

Supplementary Materials for Quantifying the impact of antibiotic use and genetic determinants of resistance on bacterial lineage dynamics

David Helekal[1], Tatum D. Mortimer[2], Aditi Mukherjee[1], Gabriella Gentile[3], Adriana Le Van[4,5], Sofia Blomqvist[1], Robert A. Nicholas[3,6], Ann E. Jerse[4], Samantha G. Palace[1], Yonatan H. Grad[1*]

[1]Department of Immunology and Infectious Diseases, Harvard T. H. Chan School of Public Health, Boston, MA 02115, USA
[2]Department of Population Health, College of Veterinary Medicine, University of Georgia, Athens, GA 30602, USA
[3]Department of Pharmacology, University of North Carolina at Chapel Hill, Chapel Hill, NC 27599, USA
[4]Department of Microbiology and Immunology, Uniformed Services University of the Health Sciences, Bethesda, MD 20814, USA
[5]Henry M. Jackson Foundation for the Advancement of Military Medicine, Inc, Bethesda, MD 20817, USA
[6]Departments of Microbiology and Immunology, University of North Carolina at Chapel Hill, Chapel Hill, NC 27599, USA

[*]Corresponding author. Email: ygrad@hsph.harvard.edu

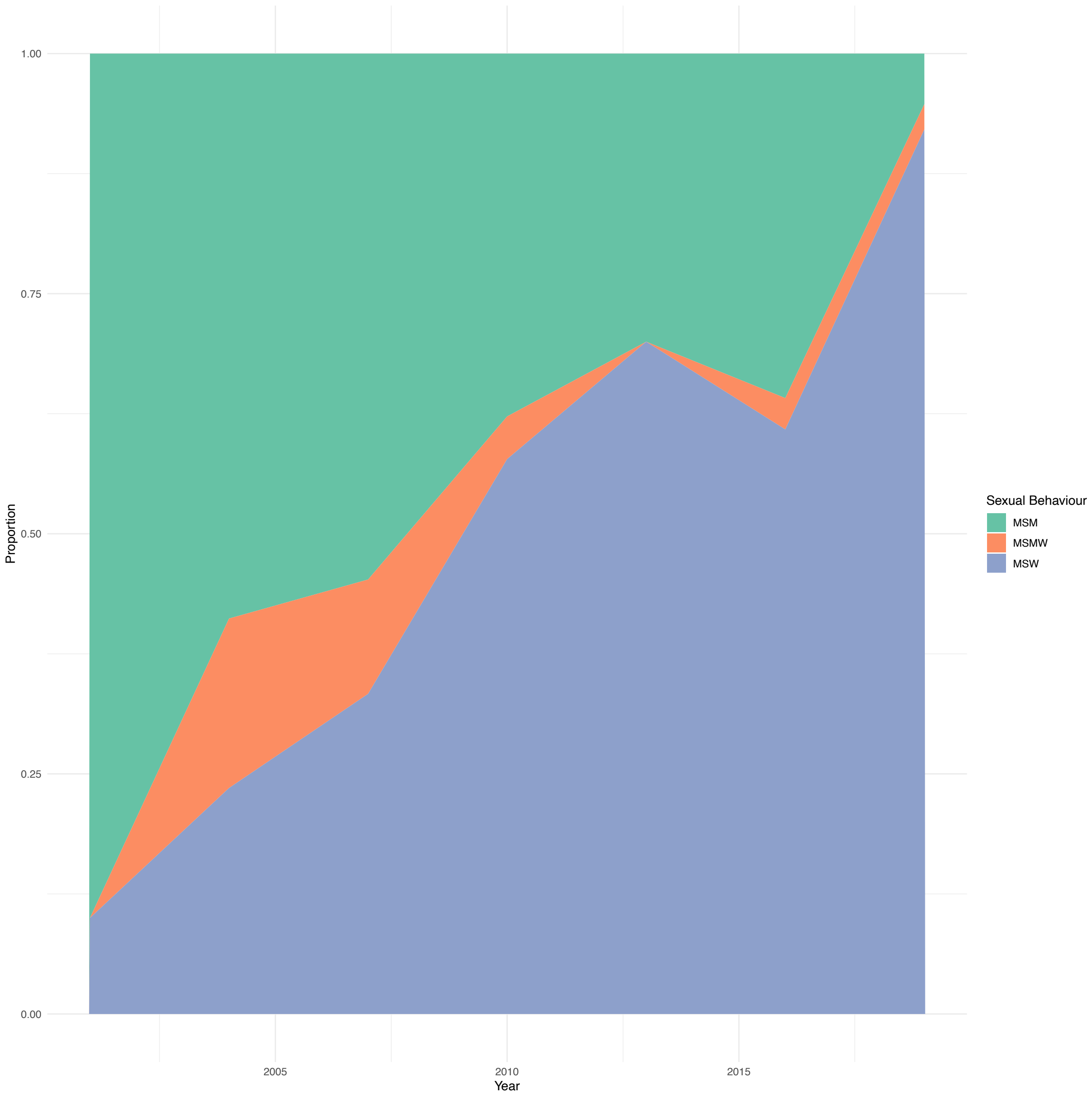

Figure S1: **Distribution of reported sexual behaviour corresponding to all descendants of lineage 20**. Proportions are calculated over 3 year intervals.

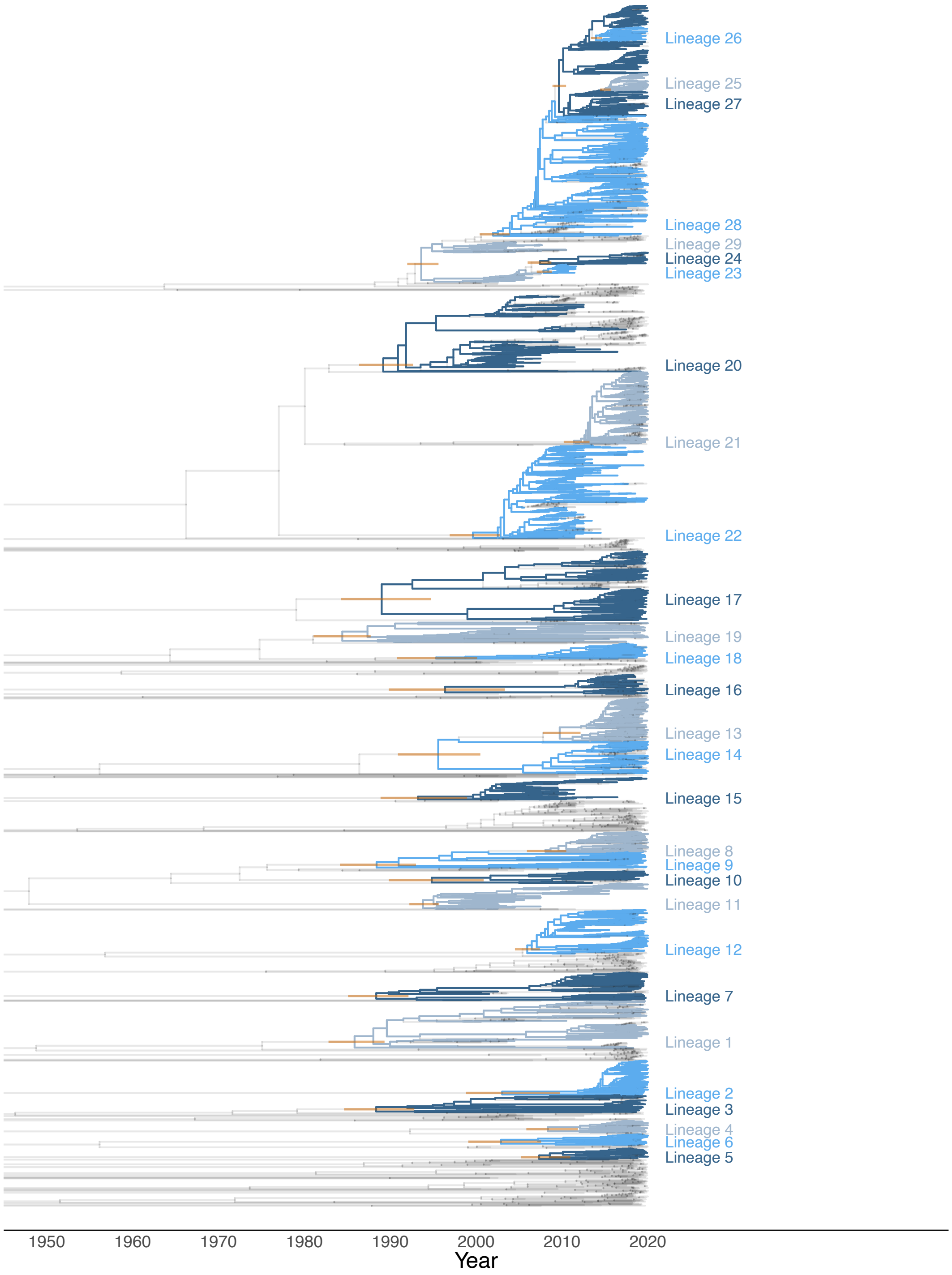

Figure S2: **Lineage assignment based on AMR determinants with timing uncertainty.** The phylogenetic tree annotated according to the lineage assignment of each node. Orange bars indicate 95% credible intervals for the timing of lineage ancestors. Gray nodes in the phylogenetic tree denote lines of descent that are not in an assigned lineage. Lineage numbering was determined by post-order traversal of the tree. Colors are for legibility purposes only.

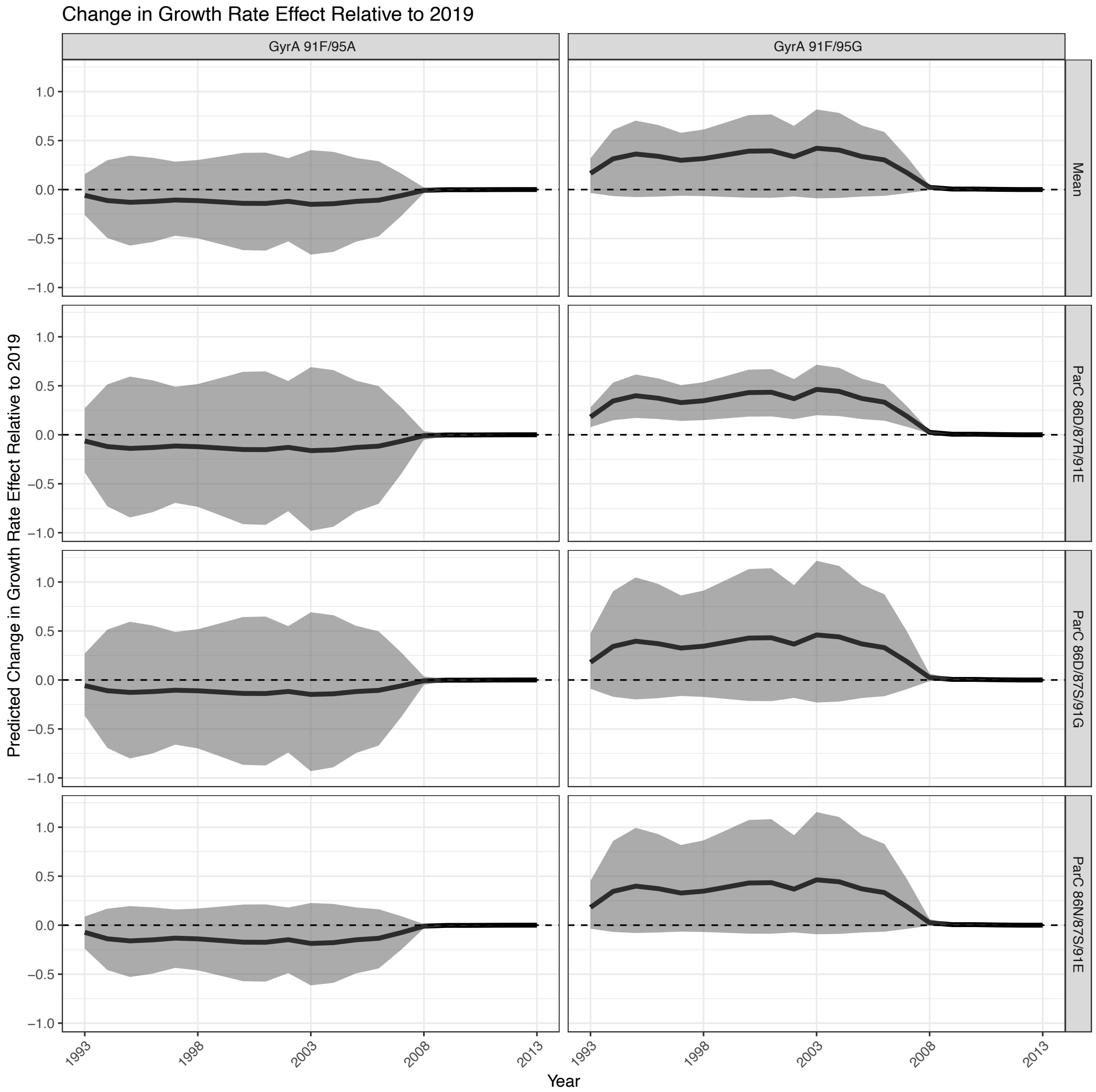

[Figure S3](#): **The predicted relative effect on growth rate for all *gyrA* determinants occurring in the dataset across all possible *parC* allele contexts**. The predicted effect shows how the growth rate effect of a given determinant has changed based on the reported treatments compared to that determinant's estimated growth rate effect in 2019. The average effect across all *parC* contexts for each of the *gyrA* alleles is denoted by Mean. The shaded region denotes the 95% posterior credible interval around the posterior median, depicted by the bold black line. Dashed line denotes no change in predicted effect of a given determinant compared to its' predicted effect in 2019.

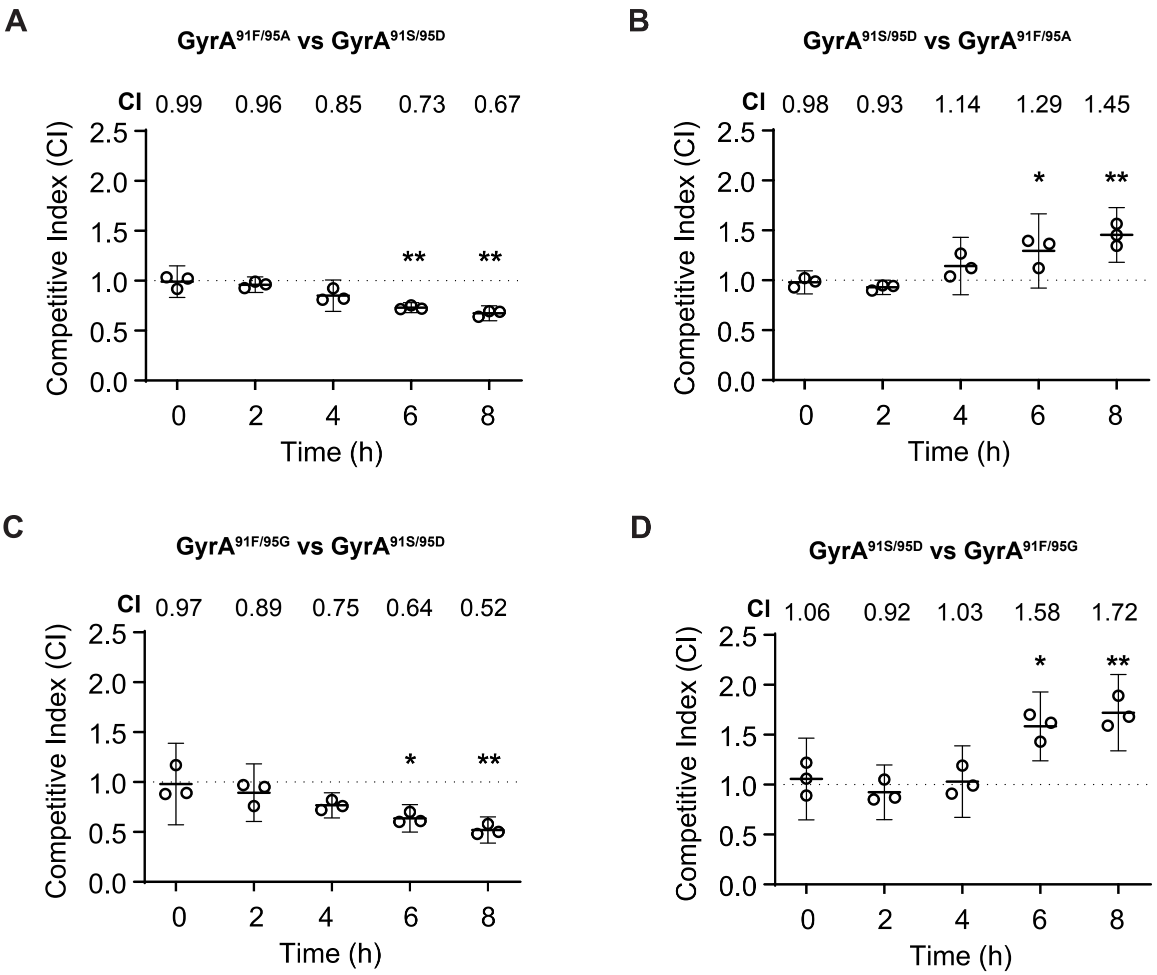

Figure S4: ***In vitro* competition assays between GCGS0481 GyrA 91S/95D, GyrA 91F/95A and GyrA 91F/95G in the ParC 86D/87R/91E context.** Panel A: Competition between unlabeled GCGS0481 GyrA 91S/95D and kanamycin-labeled GyrA 91F/95A. Statistical significance (for 2, 4, 6 and 8 hours, p = 0.49, 0.05, 0.0025, 0.0015 respectively). Panel B: Competition between unlabeled GCGS0481 GyrA 91F/95A and kanamycin-labeled GyrA 91S/95D. Statistical significance (for 2, 4, 6 and 8 hours, p = 0.19, 0.09, 0.03, 0.0023 respectively). Panel C: Competition between unlabeled GCGS0481 GyrA 91S/95D and kanamycin-labeled GyrA 91F/95G. Statistical significance (for 2, 4, 6 and 8 hours, p = 0.5, 0.098, 0.03, 0.01 respectively). Panel D: Competition between unlabeled GCGS0481 GyrA 91F/95G and kanamycin-labeled GyrA 91S/95D. Statistical significance (for 2, 4, 6 and 8 hours, p = 0.31, 0.84, 0.01, 0.007 respectively). N = 3/timepoint, representative of three independent experiments performed in absence of any antibiotic pressure. Error bars represent mean with 95% CI. Statistically significant differences in CI values were analyzed using an unpaired two-sided Student's *t-test* and are indicated ($*p < 0.05$, $**p < 0.005$ and $***p < 0.0005$).

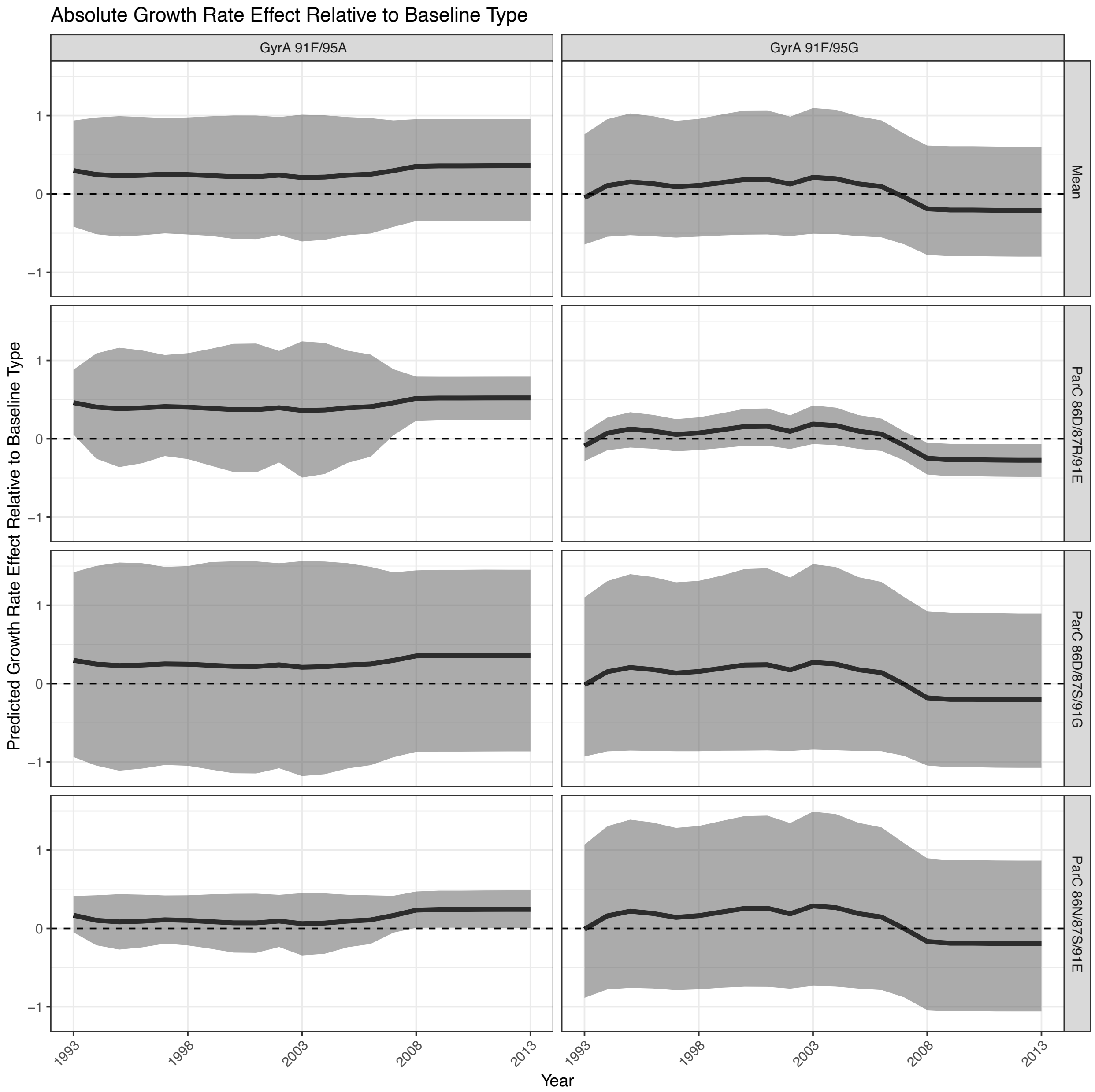

Figure S5: The predicted the absolute growth rate effect for all *gyrA* determinants occurring in the dataset across all possible *parC* allele contexts. The predicted effect was computed based on reported treatments. The average effect across all *parC* contexts for each of the *gyrA* alleles is denoted by Mean. The shaded region denotes the 95% posterior credible interval around the posterior median, depicted by the bold black line. Dashed line denotes no predicted growth rate effect relative to the baseline type that does not carry any of the resistance determinants studied.

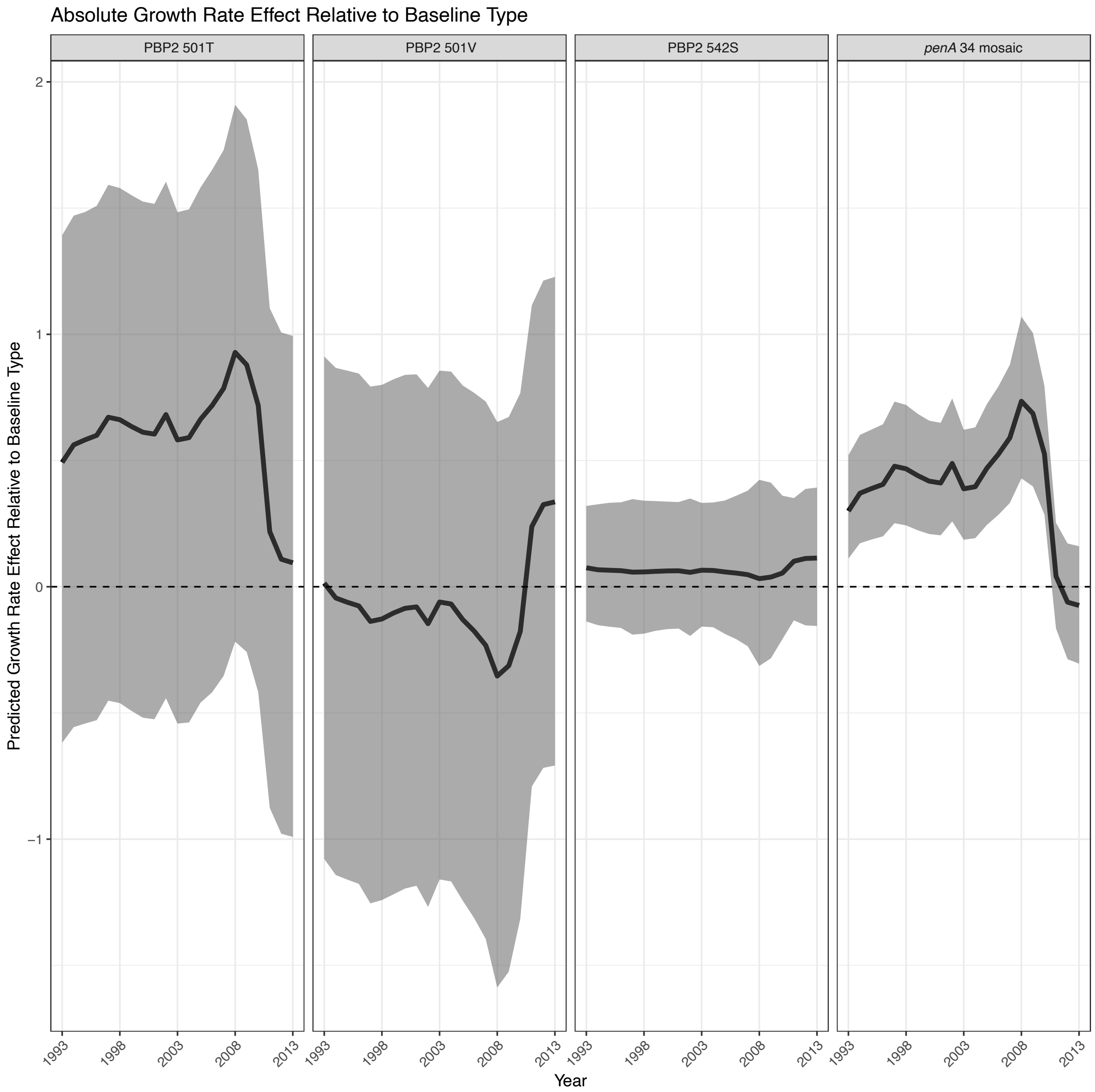

Figure S6: **The predicted the absolute growth rate effect for all determinants at the *penA* locus occurring in the dataset on lineage growth rate.** The predicted effect was computed based on reported treatments. Shaded region denotes the 95% posterior credible interval around the median, depicted by the bold black line. Dashed line denotes no predicted growth rate effect relative to the baseline type that does not carry any of the resistance determinants studied.

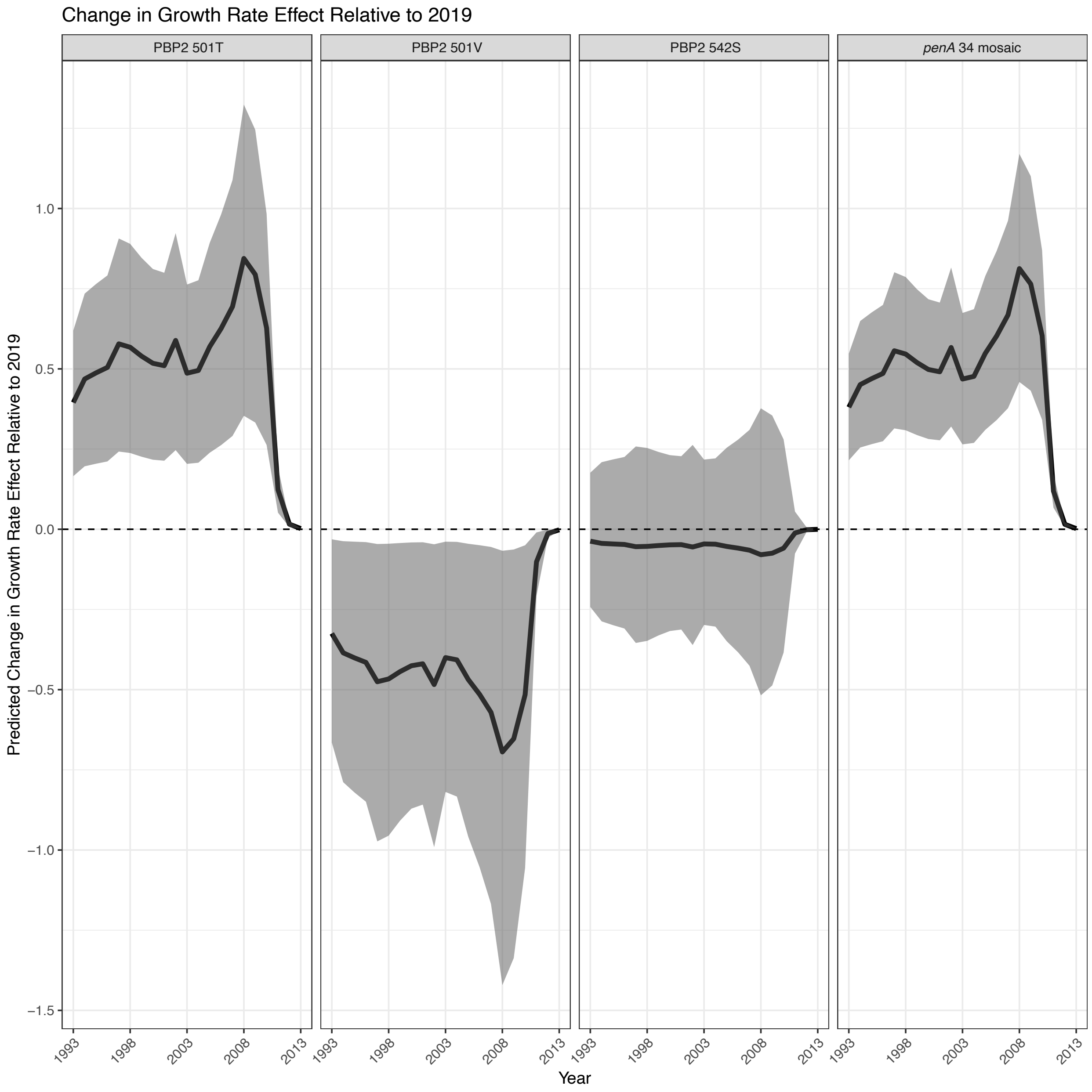

Figure S7: **The predicted relative effect on growth rate for all determinants at the *penA* locus occurring in the dataset.** The predicted effect shows how the growth rate effect of a given determinant has changed based on the reported treatments compared to that determinant's estimated growth rate effect in 2019. Shaded region denotes the 95% posterior credible interval around the median, depicted by the bold black line. Dashed line denotes no change in predicted effect of a given determinant compared to its' predicted effect in 2019.

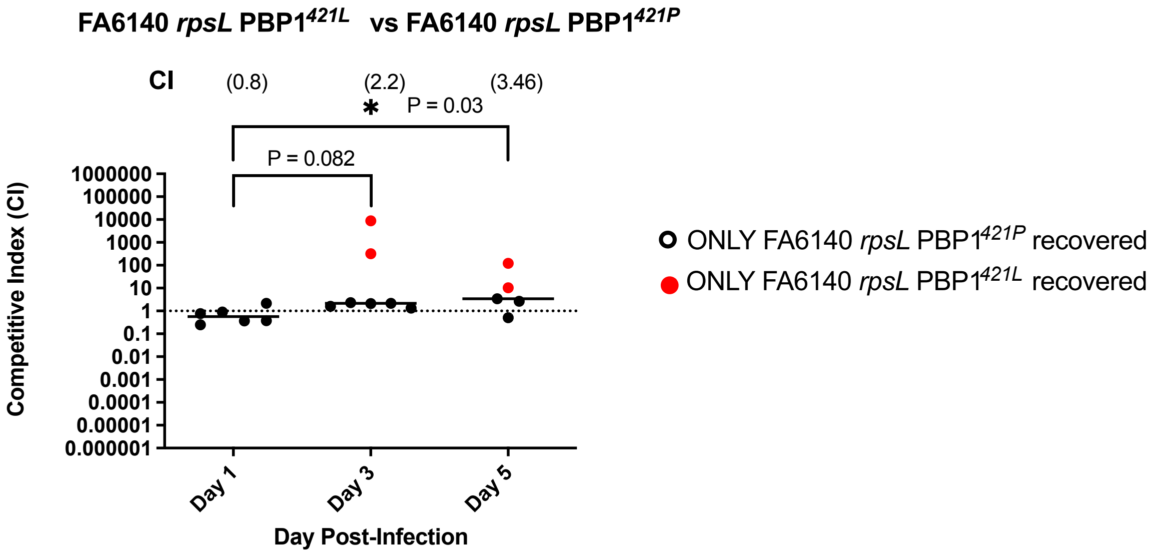

[Figure S8]: ***In vivo* murine competition assay between FA6140 *rpsL* PBP1^{421L} vs FA6140 *rpsL* PBP1^{421P}.** Measurements were taken at three time points: 1, 3, and 5 days post-infection. N = 7 mice/time point from a single infection experiment. Each point represents the CI measurement for an individual mouse. The horizontal lines indicate the geometric mean of the CI, also shown in parentheses. Statistical significance of CI measurements was assessed using the two-sided Mann-Whitney test ($*p < 0.05$, $**p < 0.005$ and $***p < 0.0005$).

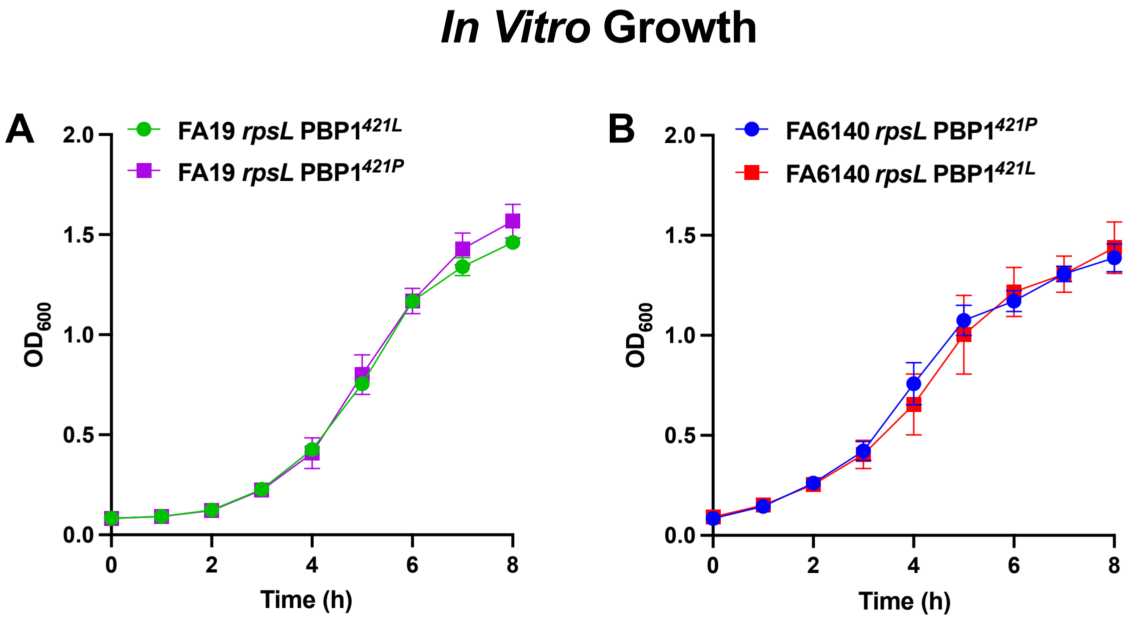

[Figure S9]: ***In vitro* growth phenotypes of isogenic PBP1 421L and PBP1 421P strain pairs grown in monoculture.** (A) Growth curves for strains FA19 *rpsL* and FA19 *rpsL* PBP1^{421P}. (B) Growth curves for strains FA6140 *rpsL* and FA6140 *rpsL* PBP1^{421L}. Data are plotted as the mean OD_{600} together with the 95% CI at each time point from (N=3) independent experiments. Statistical significance was assessed using a repeated-measures 2-way ANOVA with Tukey's multiple comparisons. No significant differences were found.

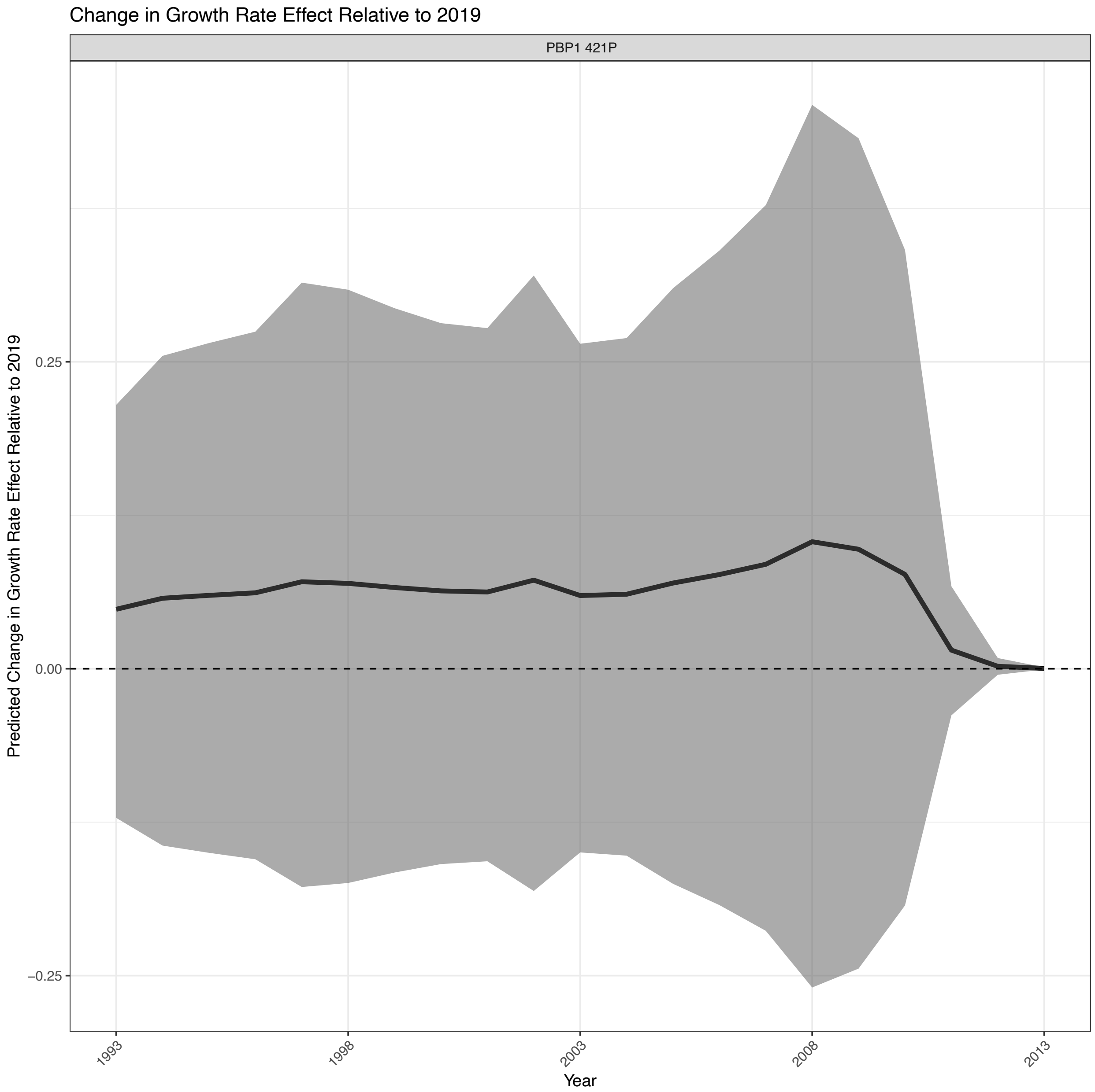

Figure S10: **The predicted relative effect on growth rate for all determinants at the *ponA* locus occurring in the dataset.** The predicted effect shows how the growth rate effect of a given determinant has changed based on the reported treatments compared to that determinant's estimated growth rate effect in 2019. Shaded region denotes the 95% posterior credible interval around the median, depicted by the bold black line. Dashed line denotes no change in predicted effect of a given determinant compared to its' predicted effect in 2019.

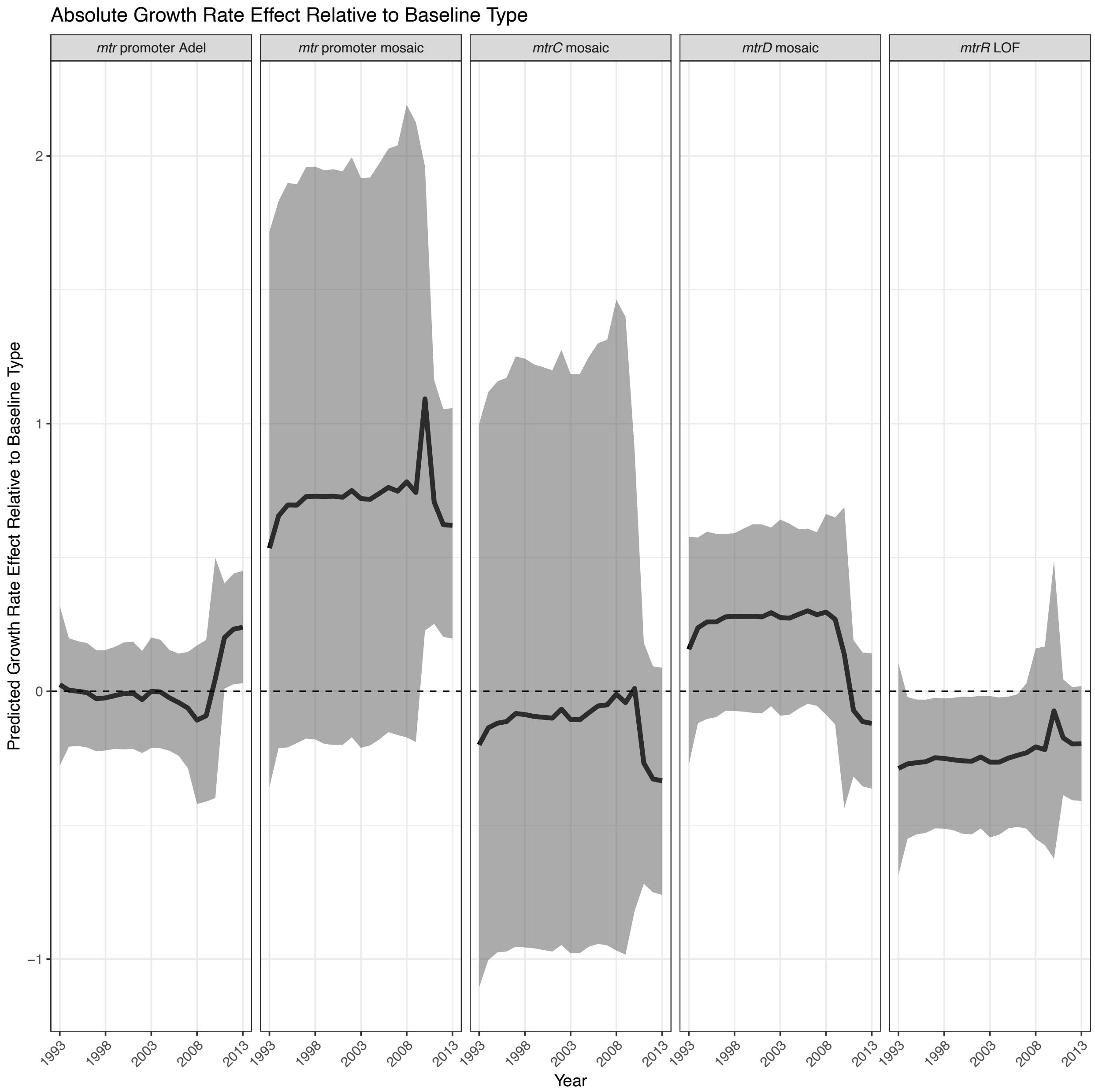

<u>Figure S11</u>: **The predicted the absolute growth rate effect for all determinants at the *mtr* operon occurring in the dataset on lineage growth rate.** The predicted effect was computed based on reported treatments. Shaded region denotes the 95% posterior credible interval around the median, depicted by the bold black line. Dashed line denotes no predicted growth rate effect relative to the baseline type that does not carry any of the resistance determinants studied.

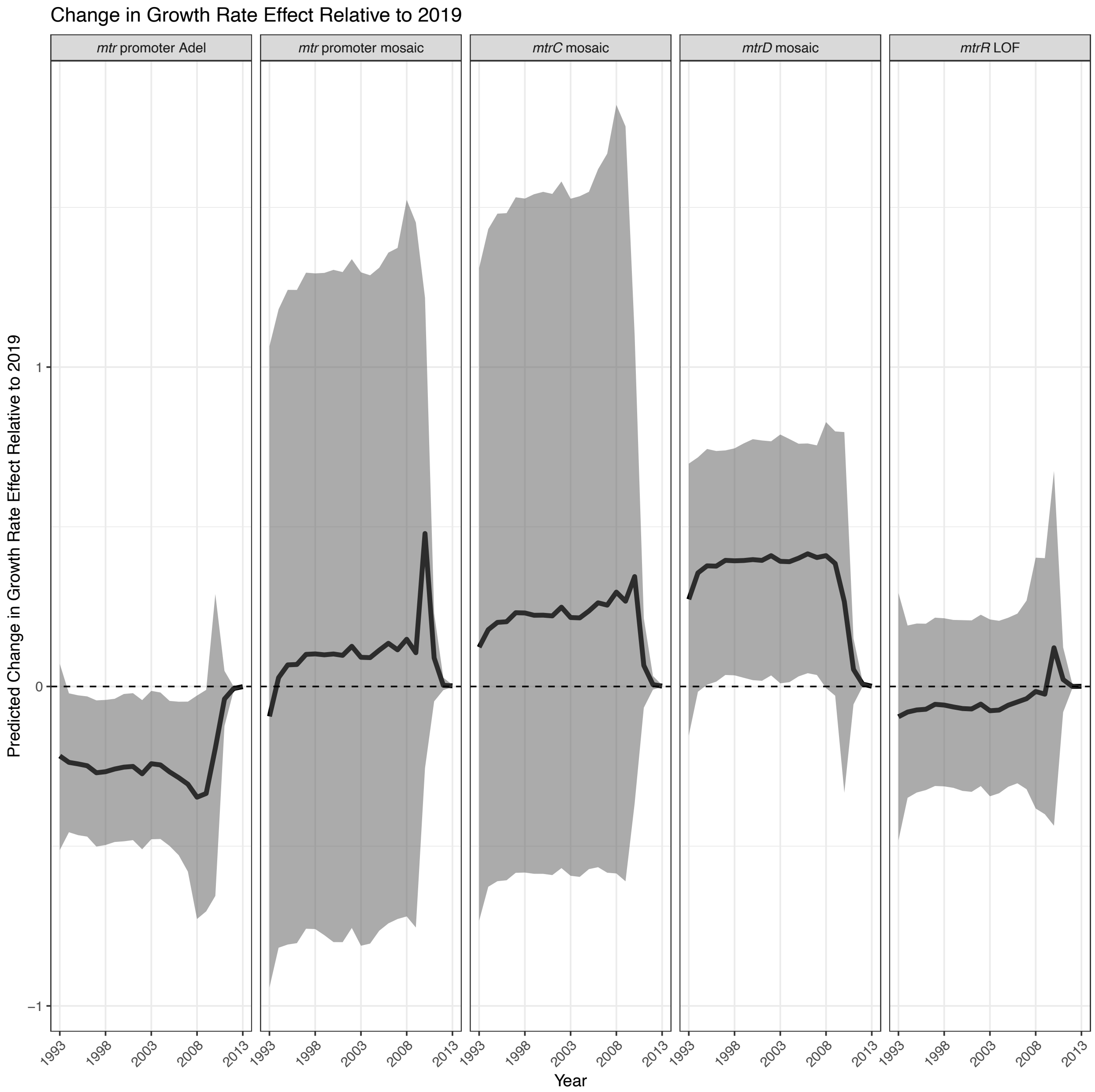

Figure S12: **The predicted relative effect on growth rate for all determinants at the *mtr* operon occurring in the dataset.** The predicted effect shows how the growth rate effect of a given determinant has changed based on the reported treatments compared to that determinant's estimated growth rate effect in 2019. Shaded region denotes the 95% posterior credible interval around the median, depicted by the bold black line. Dashed line denotes no change in predicted effect of a given determinant compared to its' predicted effect in 2019.

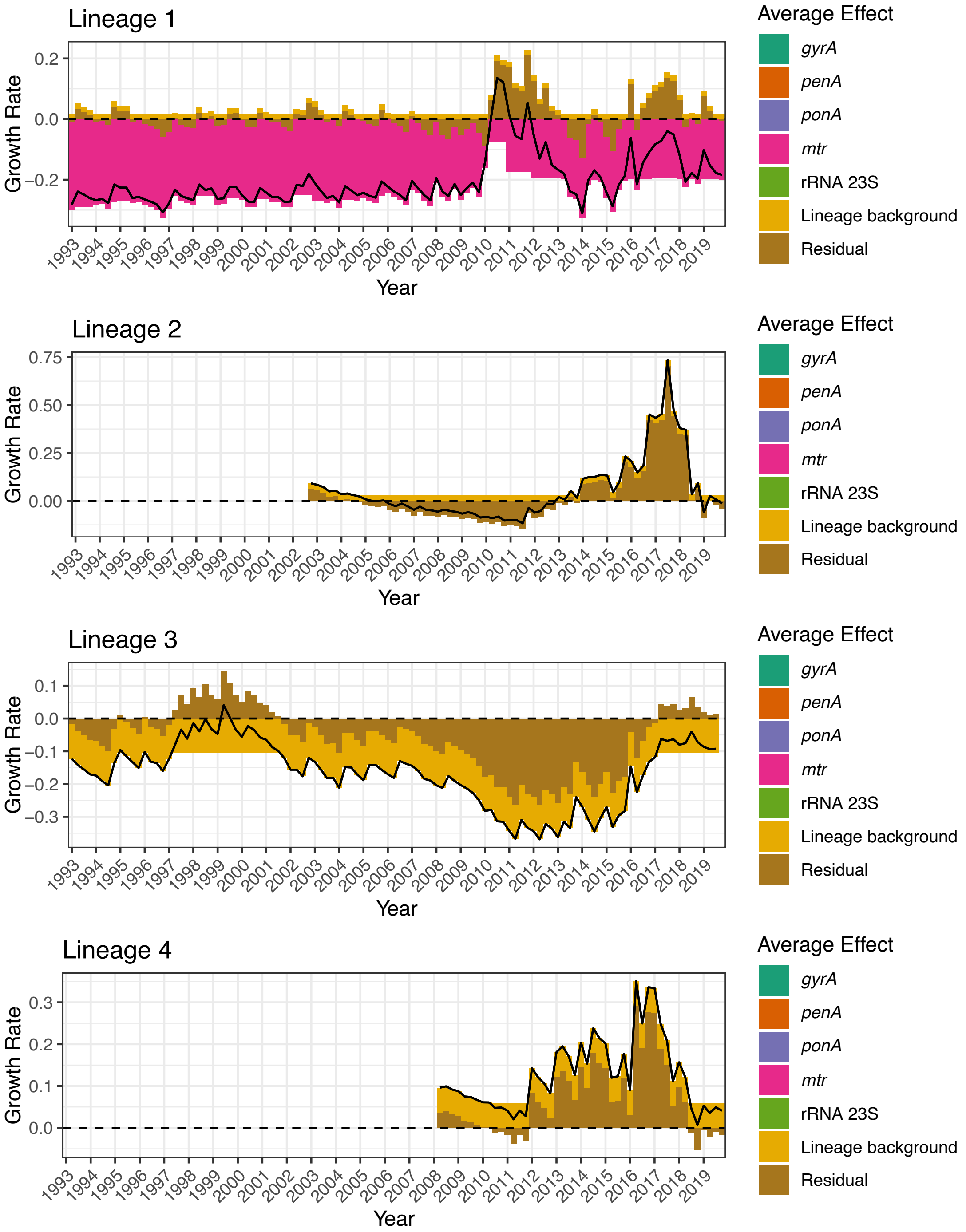

Figure S13: **Growth rate effect summary for Lineages 1-4.** The top panel shows the combined average growth rate effect of resistance determinants along with the lineage background term and the residual. The black solid line represents the total average effect. The dashed horizontal line indicates zero. Posterior summaries with uncertainty estimates for epochs spanning 1993-2007, 2007-2010, 2010-2012, and 2012-2019 available in (Supplementary Tables 1)

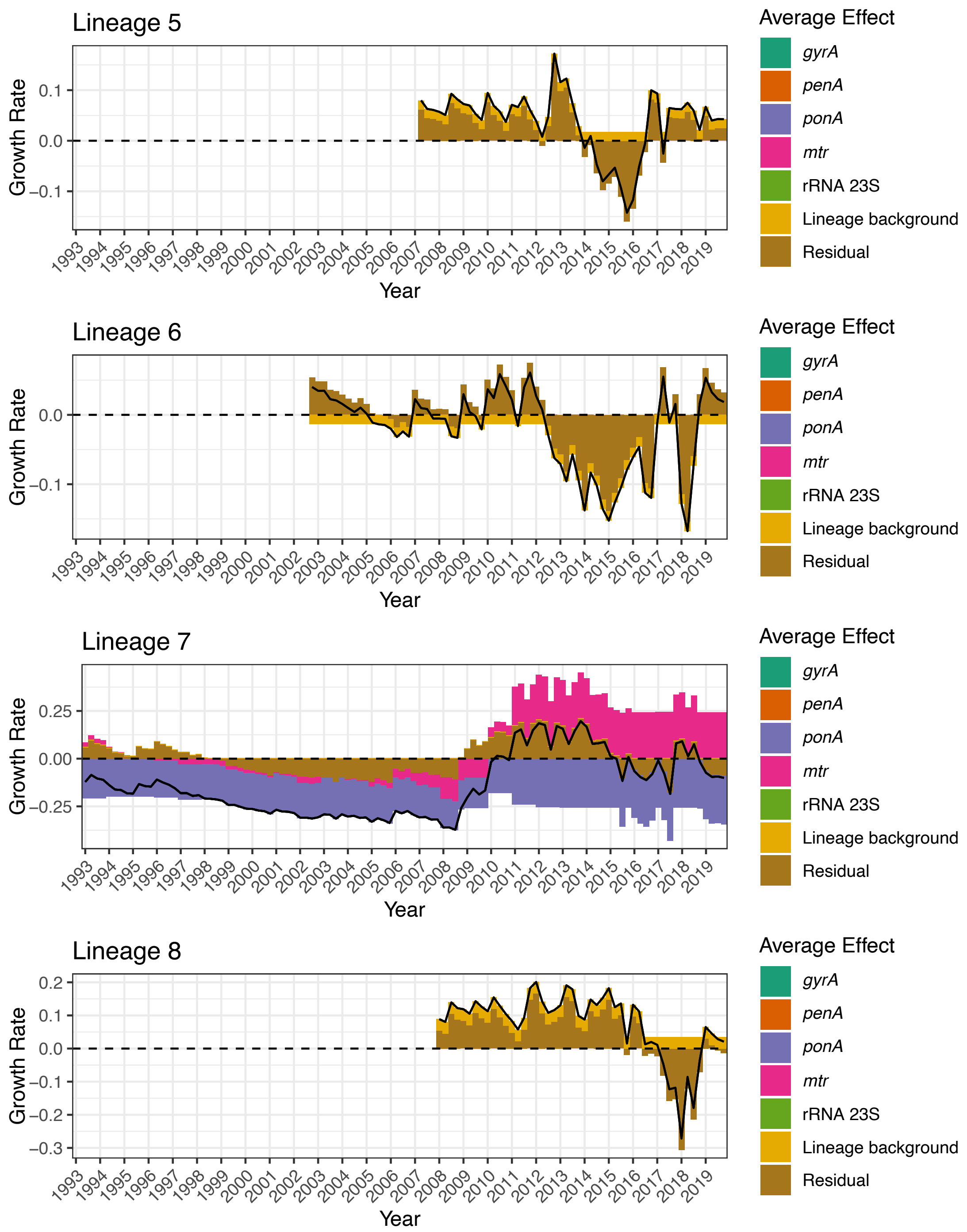

Figure S14: **Growth rate effect summary for Lineages 5-8.** The top panel shows the combined average growth rate effect of resistance determinants along with the lineage background term and the residual. The black solid line represents the total average effect. The dashed horizontal line indicates zero. Posterior summaries with uncertainty estimates for epochs spanning 1993-2007, 2007-2010, 2010-2012, and 2012-2019 available in (Supplementary Tables 1)

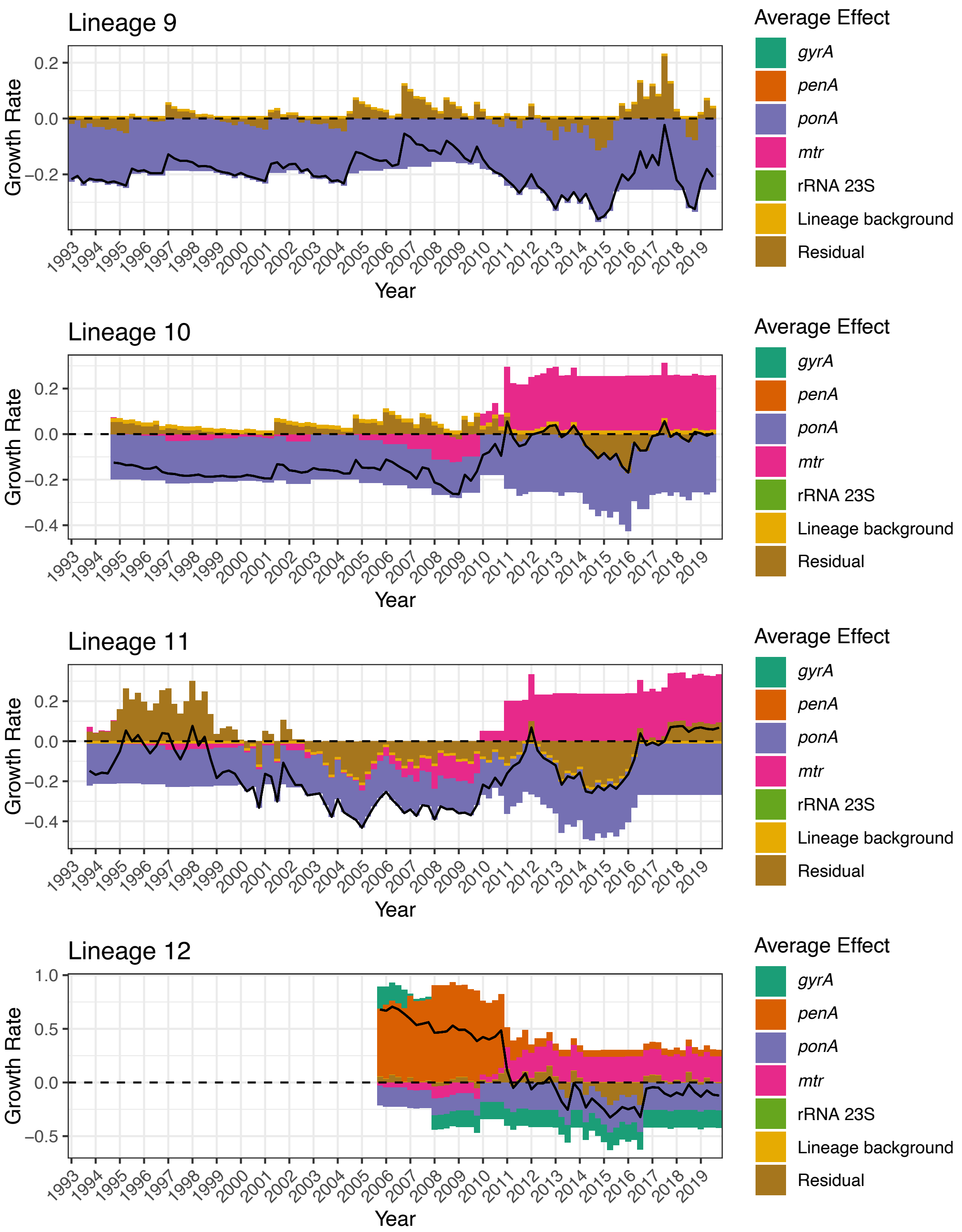

Figure S15: **Growth rate effect summary for Lineages 9-12.** The top panel shows the combined average growth rate effect of resistance determinants along with the lineage background term and the residual. The black solid line represents the total average effect. The dashed horizontal line indicates zero. Posterior summaries with uncertainty estimates for epochs spanning 1993-2007, 2007-2010, 2010-2012, and 2012-2019 available in (Supplementary Tables 1)

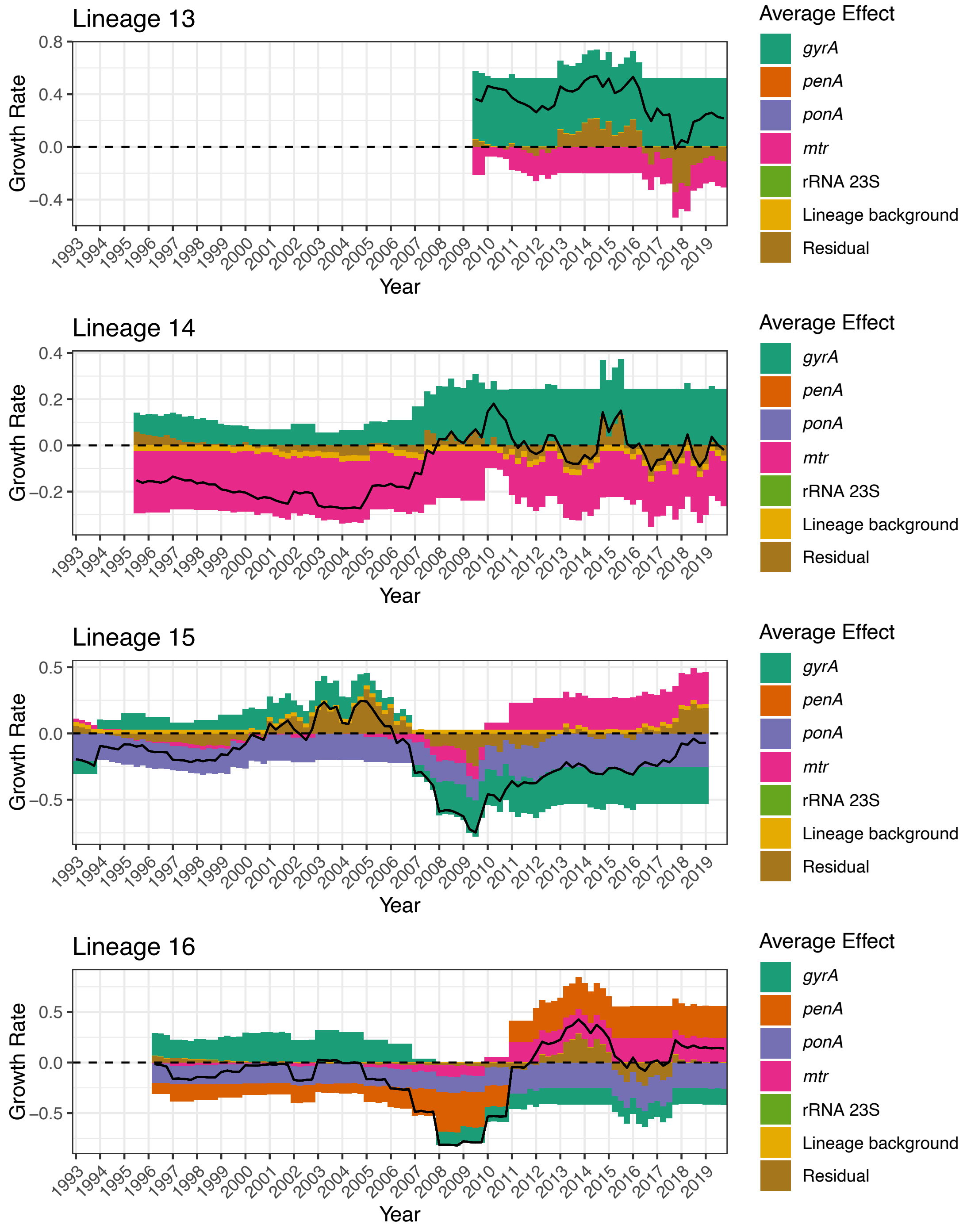

[Figure S16](#): **Growth rate effect summary for Lineages 13-16.** The top panel shows the combined average growth rate effect of resistance determinants along with the lineage background term and the residual. The black solid line represents the total average effect. The dashed horizontal line indicates zero. Posterior summaries with uncertainty estimates for epochs spanning 1993-2007, 2007-2010, 2010-2012, and 2012-2019 available in (Supplementary Tables 1)

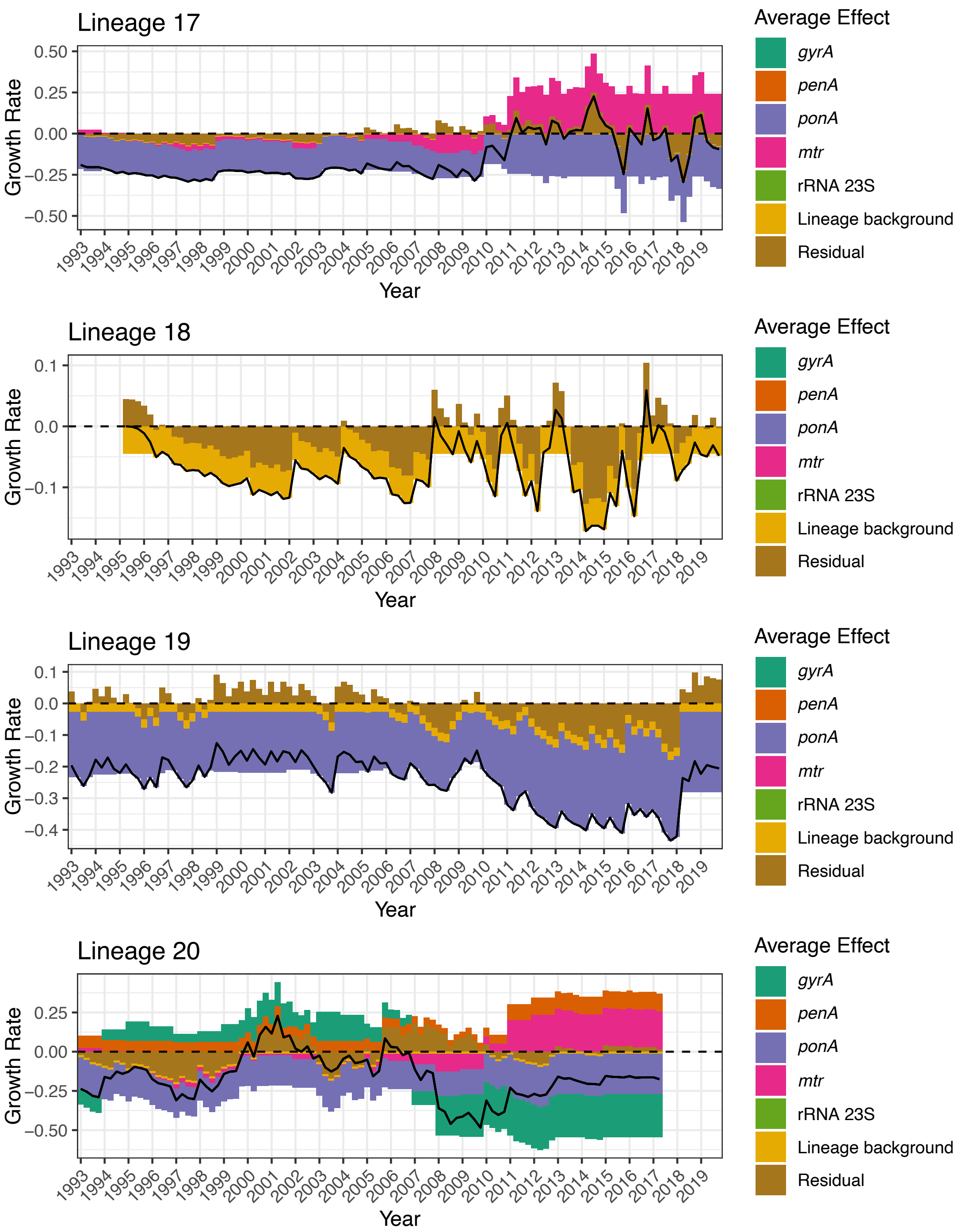

Figure S17: **Growth rate effect summary for Lineages 17-20.** The top panel shows the combined average growth rate effect of resistance determinants along with the lineage background term and the residual. The black solid line represents the total average effect. The dashed horizontal line indicates zero. Posterior summaries with uncertainty estimates for epochs spanning 1993-2007, 2007-2010, 2010-2012, and 2012-2019 available in (Supplementary Tables 1)

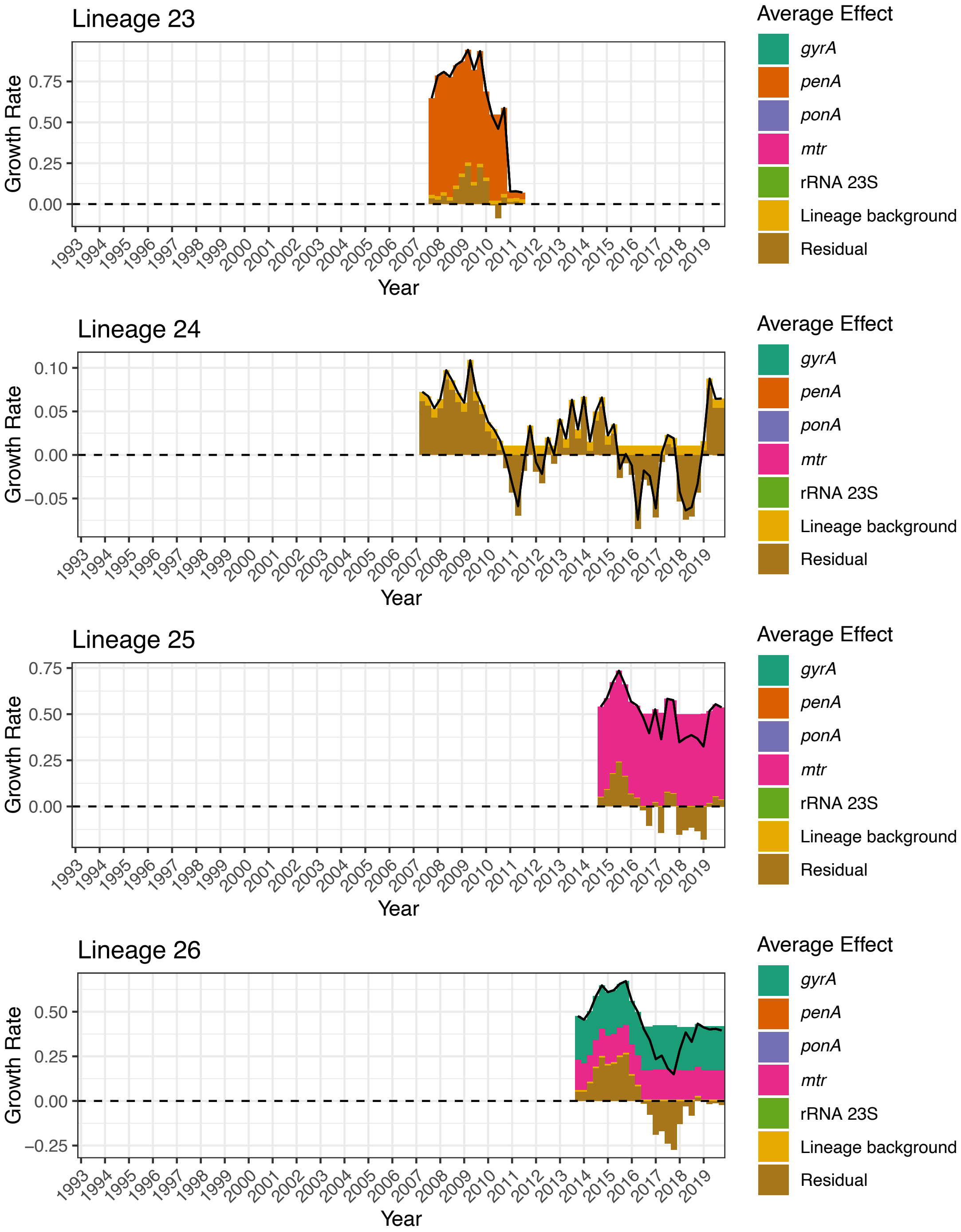

Figure S18: **Growth rate effect summary for Lineages 23-26.** The top panel shows the combined average growth rate effect of resistance determinants along with the lineage background term and the residual. The black solid line represents the total average effect. The dashed horizontal line indicates zero. Posterior summaries with uncertainty estimates for epochs spanning 1993-2007, 2007-2010, 2010-2012, and 2012-2019 available in (Supplementary Tables 1)

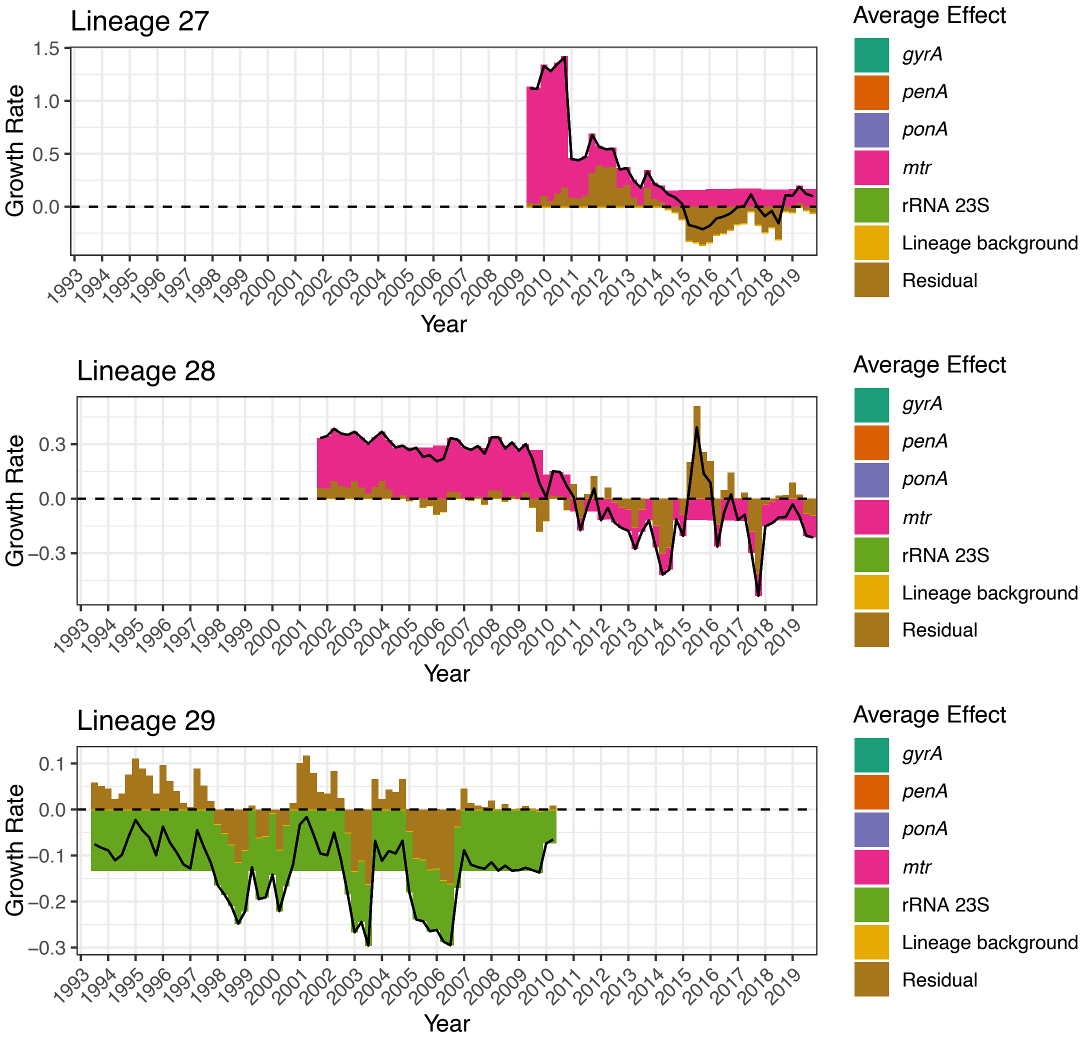

Figure S19: **Growth rate effect summary for Lineages 27-29.** The top panel shows the combined average growth rate effect of resistance determinants along with the lineage background term and the residual. The black solid line represents the total average effect. The dashed horizontal line indicates zero. Posterior summaries with uncertainty estimates for epochs spanning 1993-2007, 2007-2010, 2010-2012, and 2012-2019 available in (Supplementary Tables 1)

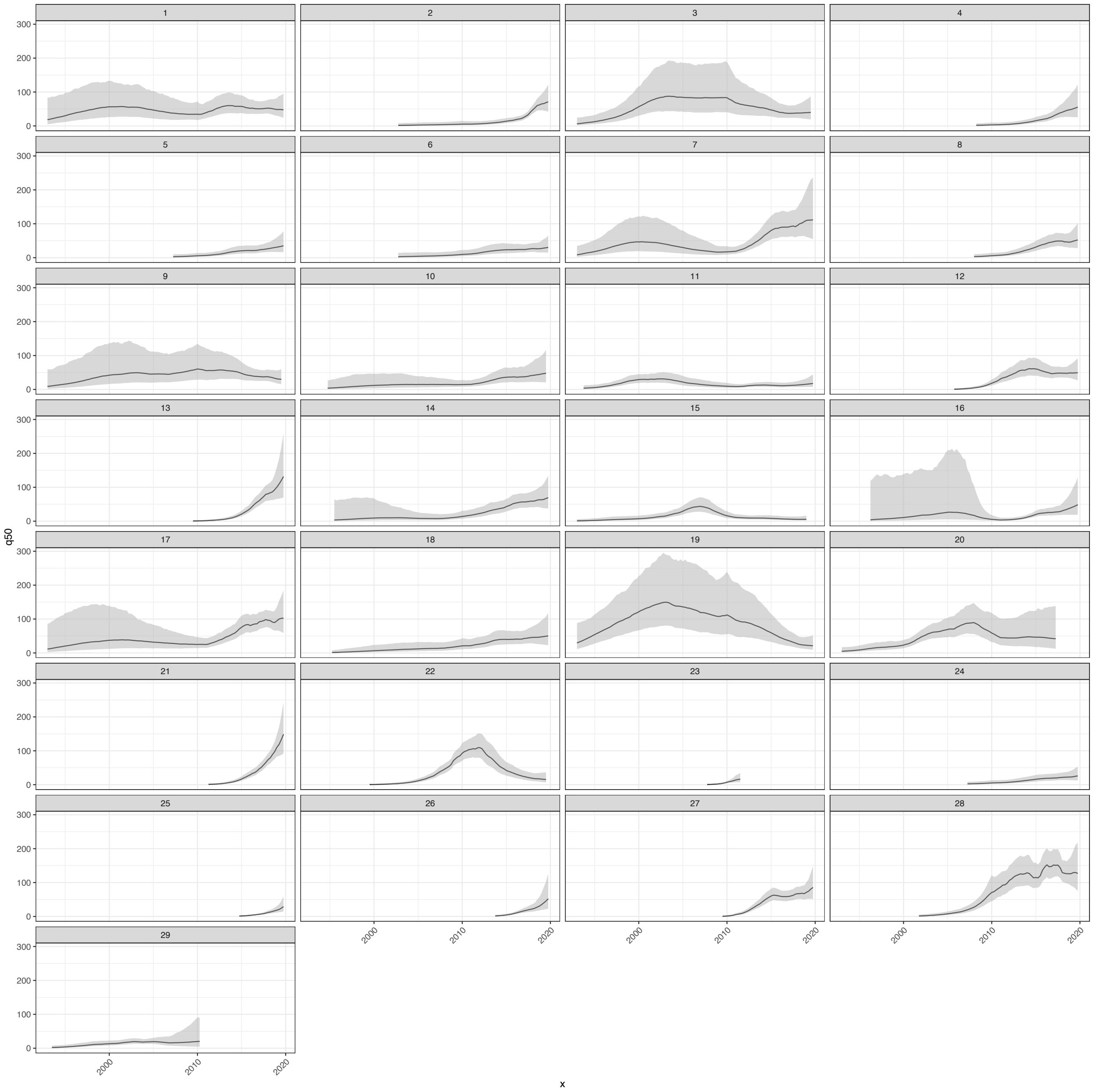

[Figure S20](#): **Estimated Ne(t) trajectories for each of the 29 lineages included in the model.** The trajectories include the residual term. The trajectories are truncated past the respective lineage MRCA.

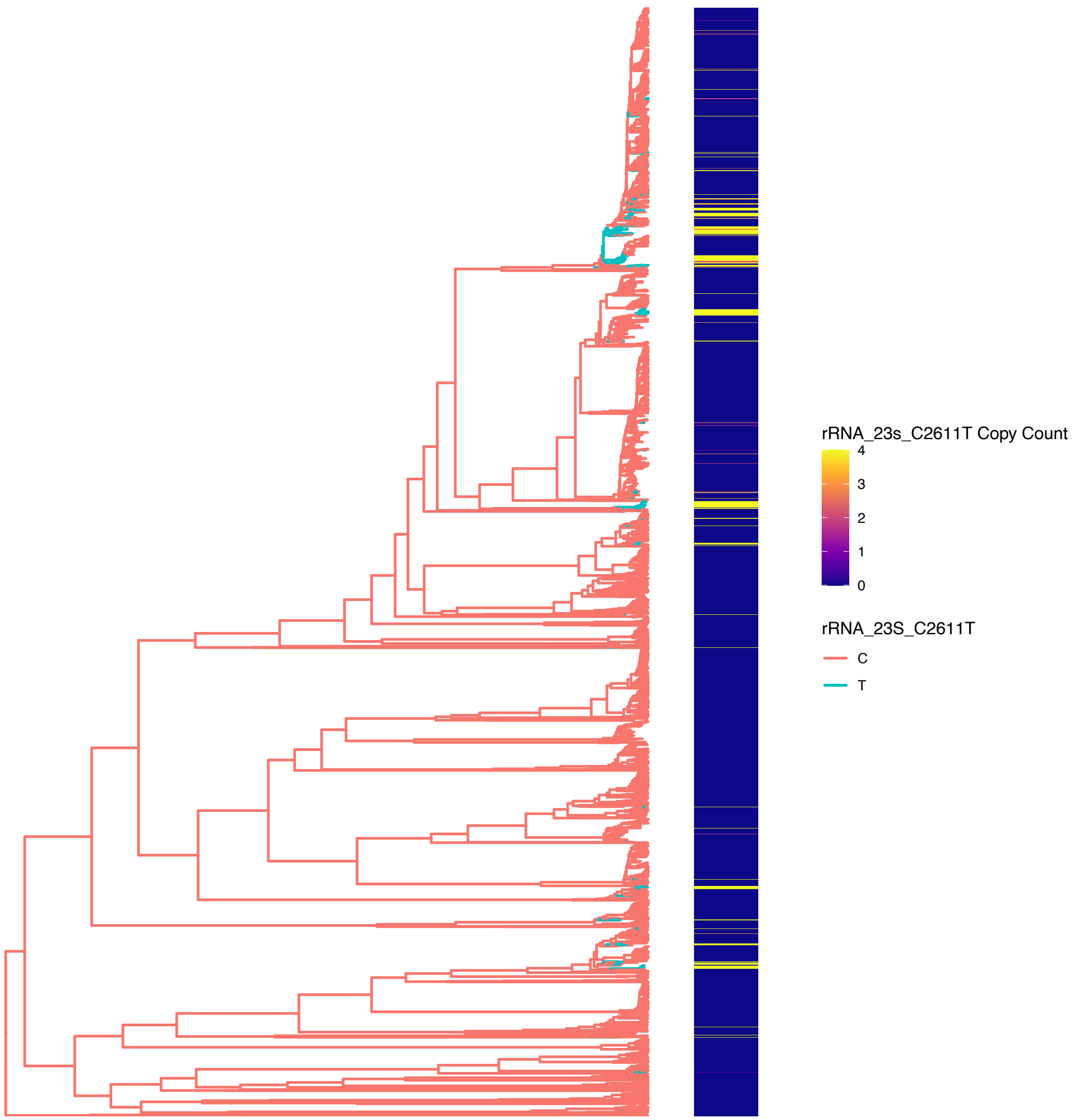

Figure S21: **Distribution of 23S rRNA C2611T across the phylogenetic tree.** The tree coloring corresponds to a DELTRAN based ancestral state reconstruction.

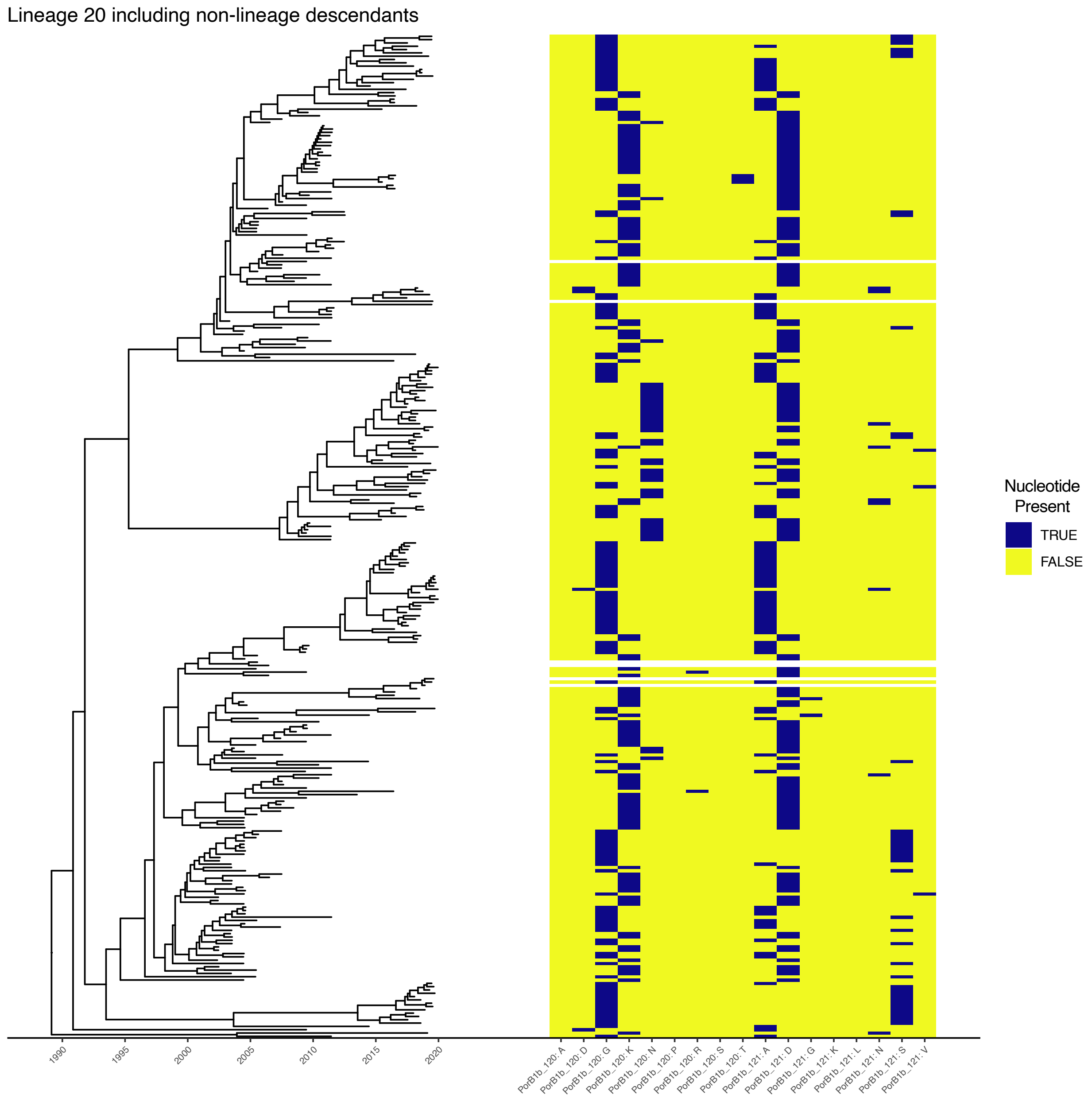

Figure S22: **The distribution of PorB G120 and A121 polymorphisms in lineage 20.** Note the high rate of loss and gain of polymorphisms and lack of clonal inheritance.

Table S1: **Interactions between individual loci and antibiotics classes as coded in the regression model used.**

Locus	Fluoroquinolones	Cephalosporins other than Cextriaxone 250mg	Azithromycin
gyrA	Yes	No	No
ponA	No	Yes	No
penA	No	Yes	No
mtr	Yes	Yes	Yes
23S rRNA	No	No	Yes

Table S2: **Predicted effect summaries (Posterior median and in brackets 95% posterior credible interval) for all the determinants included in the model.** The predicted effect summary is computed as the given effect averaged over either the in-use time period or the out-of-use time period. The average change in relative effect is computed as

the change in effect relative to 2019 averaged over the in-use period. For *gyrA* the in-use period covers 1993-2006, and the out-of-use period covers 2007 onward. For *penA*, *ponA*, *mtrCDE*, and rRNA 23S the in-use period covers 1993-2011, and the out-of-use period covers 2012 onwards..

	**Level**	**Predicted Average Effect In Use**	**Predicted Average Effect Out of Use**	**Average Change in Predicted Effect Relative to 2019**
**gyrA**				
gyrA 91F/95A	Mean	0.24 (-0.53, 0.98)	0.35 (-0.35, 0.96)	-0.12 (-0.53, 0.32)
gyrA 91F/95A	parC 86D/87R/91E	0.40 (-0.31, 1.13)	0.52 (0.23, 0.79)	-0.13 (-0.79, 0.55)
gyrA 91F/95A	parC 86D/87S/91G	0.24 (-1.08, 1.54)	0.36 (-0.87, 1.45)	-0.12 (-0.75, 0.56)
gyrA 91F/95A	parC 86N/87S/91E	0.09 (-0.24, 0.43)	0.24 (0.01, 0.48)	-0.15 (-0.49, 0.18)
gyrA 91F/95G	Mean	0.13 (-0.54, 0.99)	-0.20 (-0.78, 0.62)	0.34 (-0.07, 0.66)
gyrA 91F/95G	parC 86D/87R/91E	0.10 (-0.13, 0.30)	-0.26 (-0.47, -0.05)	0.37 (0.16, 0.57)
gyrA 91F/95G	parC 86D/87S/91G	0.18 (-0.86, 1.36)	-0.19 (-1.05, 0.92)	0.37 (-0.18, 0.98)
gyrA 91F/95G	parC 86N/87S/91E	0.19 (-0.77, 1.35)	-0.17 (-1.05, 0.89)	0.37 (-0.07, 0.93)
**penA**				
penA 501T	Mean	0.64 (-0.48, 1.56)	0.10 (-0.99, 0.99)	0.55 (0.23, 0.86)
penA 501V	Mean	-0.11 (-1.23, 0.81)	0.34 (-0.71, 1.23)	-0.45 (-0.92, -0.04)
penA 543S	Mean	0.06 (-0.18, 0.34)	0.11 (-0.15, 0.39)	-0.05 (-0.34, 0.24)
penA mosaic	Mean	0.45 (0.23, 0.70)	-0.07 (-0.30, 0.16)	0.53 (0.30, 0.76)
**ponA**				
ponA 421P	Mean	-0.19 (-0.39, -0.01)	-0.26 (-0.46, -0.05)	0.07 (-0.17, 0.30)
**mtrCDE**				
mtr promoter Adel	Mean	-0.01 (-0.20, 0.16)	0.24 (0.03, 0.45)	-0.25 (-0.47, -0.04)
mtr promoter mosaic	Mean	0.74 (-0.10, 1.88)	0.62 (0.19, 1.05)	0.11 (-0.67, 1.19)
mtrC mosaic	Mean	-0.09 (-0.91, 1.12)	-0.33 (-0.76, 0.08)	0.22 (-0.51, 1.43)
mtrD mosaic	Mean	0.25 (-0.07, 0.53)	-0.12 (-0.36, 0.14)	0.36 (0.03, 0.67)
mtrR LOF	Mean	-0.23 (-0.49, -0.03)	-0.20 (-0.41, 0.02)	-0.05 (-0.27, 0.20)
**rRNA 23S**				
rRNA 23s 2611T	Mean	-0.12 (-0.40, 0.11)	-0.07 (-0.40, 0.26)	-0.06 (-0.40, 0.31)

Table S3: **Ciprofloxacin Minimum Inhibitory Concentrations (MICs) of *N. gonorrhoeae* isogenic strains**

**Strains**	**Ciprofloxacin MIC (μg/mL)**
GCGS0481 GyrA 91F/95G (Parent)	≥32
GCGS0481 GyrA 91S/95D, CMR	0.25

GCGS0481 GyrA 91F/95G, CMR	≥32
GCGS0481 GyrA 91F/95A, CMR	≥32

Table S4: ***N. gonorrhoeae* strains used in this study for GyrA mutant competition experiments.**

Strains	Description	Reference
GCGS0481_GyrA 91F/95G (Parent)	Clinical N. gonorrhoeae isolate GCGS0481, GyrA 91F/95G	Centers for Disease control's Gonococcal Isolate Surveillance Project (GISP)
GCGS0481_GyrA 91S/95D	Clinical isogenic N. gonorrhoeae isolate GCGS0481, CMR, GyrA 91S/95D	This study
GCGS0481_GyrA 91F/95G	Clinical isogenic N. gonorrhoeae isolate GCGS0481, CMR, GyrA 91F/95G	This study
GCGS0481_GyrA 91F/95A	Clinical isogenic N. gonorrhoeae isolate GCGS0481, CMR, GyrA 91F/95A	This study
GCGS0481_GyrA 91S/95D	Clinical isogenic N. gonorrhoeae isolate GCGS0481, CMR, KanR, GyrA 91S/95D	This study
GCGS0481_GyrA 91F/95G	Clinical isogenic N. gonorrhoeae isolate GCGS0481, CMR, KanR, GyrA 91F/95G	This study
GCGS0481_GyrA 91F/95A	Clinical isogenic N. gonorrhoeae isolate GCGS0481, CMR, KanR, GyrA 91F/95A	This study

Table S5: **Plasmids used in this study for GyrA mutant competition experiments.**

Plasmids	Description	Reference
pDRE77	pUC19 with AphA3 Kan^R cassette + homology to clone GyrA 91S/95D	(79)
pKH37	CM^R, lac-inducible, inserts into lctP/aspC site of N. gonorrhoeae	(80)
pAM_3	pUC19 with CM^R cassette + homology to clone GyrA 91S/95D	This study
pDR1	KanR derivative of pKH37	(81)
pDR53	A derivative of pDR1 without Lac promoter and the regulatory elements + added multi cloning site (MCS)	This study

Table S6: **Primers used in this study for GyrA mutants competition experiments.**

Primers	Sequence (Annealing)	Description
AM_7	GGGGCGTAATACCTAGATTTAGATGTCTAAAAAGCGTCGAC	Forward to amplify GyrA 91S/95D from pDRE77
AM_8	TTTTTTTCTCCATTTATTATTTCCTTCCTCTTTTCTACAGTATTTA	Reverse to amplify GyrA 91S/95D from pDRE77
AM_9	ATCTAGGTATTACGCCCCGCCCTG	Forward to amplify CMR cassette from pKH37
AM_10	AGGAAATAATAAATGGAGAAAAAAATCACTGGATATACCACC	Reverse to amplify CMR cassette from pKH37
AM_5	ATGCCGTCTGAAATCCCGACAAATTCGCTTC	Forward to amplify GyrA allele from Ng gDNA
AM_6	TTGCCTATGGGTTCGATATG	Reverse to amplify GyrA allele from Ng gDNA

DR_395	ATTAAATGCGTTTAAACATGCTCTAGAACGCGTCGACGCT	Forward to amplify KanR derivative of pKH37 from pDRE1
DR_396	TGTTTAAACGCATTTAATTAAGAACGGATGGTTCTTGTTG	Reverse to amplify KanR derivative of pKH37 from pDRE1
DR_63	CACCAATAACTGCCTTAAAAAAAACGCGTCGACGCTTTTTAGACATC	Forward to Sanger sequence the KanR cassette of pDR53 transformed Ng by colony PCR
DR_62	CAGGAGCTAAGGAAGCTAAACGGGATCCGCCGTCTGAAC	Reverse to Sanger sequence the KanR cassette of pDR53 transformed Ng by colony PCR

Table S7: ***N. gonorrhoeae* strains used in this study for *ponA* mutant competition experiments.** Transformants are shown as recipient strain x donor DNA, produced as described in Materials and Methods. PCR signifies a PCR product was used to transform, and pUC18 denotes all cloning plasmids used.

Strains	Description	Reference
FA19	Wild type, antibiotic-susceptible lab strain	(84)
FA6140	Penicillin-resistant clinical isolate	(35)
FA19 *rpsL* PBP1^{421L}	FA19 x rpsL1 PCR; naturally expresses PBP1 421L	(86)
FA6140 *rpsL* PBP1^{421P}	FA6140 x rpsL1 PCR; naturally expresses PBP1 421P	This study
FA19 *rpsL* PBP1^{421P}	FA19 *rpsL* x pUC18us-PBP1^{421P}-*Ω*; replaces PBP1 421L with PBP1 421P	This study
FA6140 *rpsL* PBP1^{421L}	FA6140 *rpsL* x pUC18us-PBP1^{421L}-*Ω*; replaces PBP1 421P with PBP1 421L	This study

Table S8: **Plasmids used in this study for *ponA* mutant competition experiments.**

Plasmids	Description	Reference
pUC18us-PBP1^{421P}-*Ω*	pUC18-us containing bp 831-2400 of *ponA1*, 54 bp downstream of *ponA*, the *aad1* resistance cassette (*Ω*), and 531 bp of additional downstream sequence	This study
pUC18us-PBP1^{421L}-*Ω*	pUC18-us containing bp 831-2400 of *ponA*, 54 bp downstream of *ponA*, the aad1 resistance cassette (*Ω*), and 531 bp of additional downstream sequence	This study

Table S9: **Primers used in this study for *ponA* mutant competition experiments.**

Primers	Sequence (Annealing)	Description
5'rpsL	GCCGTCTGAAATGCTTGACTGTCTGCTTGC	Forward to Sanger sequence and amplify the rpsL1 allele from Ng DNA
3'rpsL	GTCGGGTCTATTCCCATGAA	Reverse to amplify the rpsL1 allele from Ng DNA.
5'pUC18US_ponA_HiFi	TGCCAAGCTGGCCGTCTGAAAAGCTTATATGGTGAAGATGCCTATACG	Forward to amplify the ponA allele from Ng DNA for ligation in pUC18(*Ω*)
3' pUC18US_ponA_HiFi	AGCGTGCATAATAAGCCCTATCTAGAGCCAAATCTAAAATGCCGTC	Reverse to amplify the ponA allele from Ng

		DNA for ligation in pUC18(Ω)
5’ponA956	GCGGTGCGGAAAACTATATC	Forward to Sanger sequence the ponA allele of pUC18USΩ transformed Ng
5’pUC18_40	GCCAGGGTTTTCCCAGTCACGA	Forward to Sanger sequence pUC18USΩ cloning plasmids
3’pUC18_Rev	GAGCGGATAACAATTTCACACAGG	Reverse to Sanger sequence pUC18USΩ cloning plasmids
