# Supplementary material for: Quantifying the real-world impact of antibiotic use and genetic determinants of resistance on gonococcal dynamics
Source: Nat Microbiol. 2026 Jan 30;11(2):375–90. doi: 10.1038/s41564-025-02235-w (PMC12872460; doi:10.1038/s41564-025-02235-w)
Supplement: Supplementary file 2 — Reporting Summary [file 41564_2025_2235_MOESM2_ESM.pdf]

nature portfolio

Corresponding author(s): Yonatan Grad

Last updated by author(s): Dec 2, 2025

Reporting Summary

Nature Portfolio wishes to improve the reproducibility of the work that we publish. This form provides structure for consistency and transparency in reporting. For further information on Nature Portfolio policies, see our Editorial Policies and the Editorial Policy Checklist.

Statistics

For all statistical analyses, confirm that the following items are present in the figure legend, table legend, main text, or Methods section.

n/a	Confirmed	
☐	☒	The exact sample size (*n*) for each experimental group/condition, given as a discrete number and unit of measurement
☐	☒	A statement on whether measurements were taken from distinct samples or whether the same sample was measured repeatedly
☐	☒	The statistical test(s) used AND whether they are one- or two-sided<br>*Only common tests should be described solely by name; describe more complex techniques in the Methods section.*
☐	☒	A description of all covariates tested
☐	☒	A description of any assumptions or corrections, such as tests of normality and adjustment for multiple comparisons
☐	☒	A full description of the statistical parameters including central tendency (e.g. means) or other basic estimates (e.g. regression coefficient) AND variation (e.g. standard deviation) or associated estimates of uncertainty (e.g. confidence intervals)
☐	☒	For null hypothesis testing, the test statistic (e.g. *F*, *t*, *r*) with confidence intervals, effect sizes, degrees of freedom and *P* value noted<br>*Give P values as exact values whenever suitable.*
☐	☒	For Bayesian analysis, information on the choice of priors and Markov chain Monte Carlo settings
☒	☐	For hierarchical and complex designs, identification of the appropriate level for tests and full reporting of outcomes
☒	☐	Estimates of effect sizes (e.g. Cohen's *d*, Pearson's *r*), indicating how they were calculated

Our web collection on statistics for biologists contains articles on many of the points above.

Software and code

Policy information about availability of computer code

Data collection	No software was used for data collection
Data analysis	Analysis of experimental data was carried out in Graphpad Prism. Open-source software required for genomic analysis is described in Materials and Methods. Software required to reproduce the computational analysis is available at https://github.com/gradlab/GC_AMR_Lineages<br>Software used for genomic analysis:<br>- Gubbins 3.4.3<br>- IQTree 2.4.0<br>- SPAdes 3.12.0<br>- BWA-MEM 0.7.17<br>- Pilon 1.23<br>- samtools 1.17<br>- blastn 2.9.0<br>- BactDating<br>- PastML web version

For manuscripts utilizing custom algorithms or software that are central to the research but not yet described in published literature, software must be made available to editors and reviewers. We strongly encourage code deposition in a community repository (e.g. GitHub). See the Nature Portfolio guidelines for submitting code & software for further information.

Data

Policy information about availability of data

All manuscripts must include a data availability statement. This statement should provide the following information, where applicable:

- Accession codes, unique identifiers, or web links for publicly available datasets
- A description of any restrictions on data availability
- For clinical datasets or third party data, please ensure that the statement adheres to our policy

Genomic data, metadata, and accession numbers are publicly available in previously published work and listed in the github repository https://github.com/gradlab/GC_AMR_Lineages. Base-called Nanopore reads for generated GyrA mutants were deposited to NCBI SRA under PRJNA1281689.

Research involving human participants, their data, or biological material

Policy information about studies with human participants or human data. See also policy information about sex, gender (identity/presentation), and sexual orientation and race, ethnicity and racism.

Reporting on sex and gender	Not applicable
Reporting on race, ethnicity, or other socially relevant groupings	Not applicable
Population characteristics	Not applicable
Recruitment	Not applicable
Ethics oversight	Not applicable

Note that full information on the approval of the study protocol must also be provided in the manuscript.

Field-specific reporting

Please select the one below that is the best fit for your research. If you are not sure, read the appropriate sections before making your selection.

☒ Life sciences ☐ Behavioural & social sciences ☐ Ecological, evolutionary & environmental sciences

For a reference copy of the document with all sections, see nature.com/documents/nr-reporting-summary-flat.pdf

Life sciences study design

All studies must disclose on these points even when the disclosure is negative.

Sample size	Sample sizes were determined based on experimental feasibility and cost considerations
Data exclusions	No data was excluded
Replication	All experiments were replicated
Randomization	No randomization was performed as the comparisons are between isogenic controls
Blinding	No blinding was performed as the comparisons are between isogenic controls

Reporting for specific materials, systems and methods

We require information from authors about some types of materials, experimental systems and methods used in many studies. Here, indicate whether each material, system or method listed is relevant to your study. If you are not sure if a list item applies to your research, read the appropriate section before selecting a response.

Materials & experimental systems

n/a	Involved in the study	
☒	☐	Antibodies
☒	☐	Eukaryotic cell lines
☒	☐	Palaeontology and archaeology
☐	☒	Animals and other organisms
☒	☐	Clinical data
☒	☐	Dual use research of concern
☒	☐	Plants

Methods

n/a	Involved in the study	
☒	☐	ChIP-seq
☒	☐	Flow cytometry
☒	☐	MRI-based neuroimaging

Animals and other research organisms

Policy information about studies involving animals; ARRIVE guidelines recommended for reporting animal research, and Sex and Gender in Research

Laboratory animals	BALB/c mice, female, 6 to 8 weeks old, National Cancer Institute<br>Humidity: 30-70%<br>Temperature: 68-79°F or 20-26°C<br>Lighting conditions: Lights on 0600; lights off: 1800
Wild animals	Not applicable
Reporting on sex	Female only, animal model is of intra-vaginal infection
Field-collected samples	Not applicable
Ethics oversight	Uniformed Services University of the Health Sciences Institutional Animal Care and Use Committee

Note that full information on the approval of the study protocol must also be provided in the manuscript.

Plants

Seed stocks	Not applicable
Novel plant genotypes	Not applicable
Authentication	Not applicable
